# Tris(ethyl­enediamine)­nickel(II) 1*H*-pyrazole-3,5-dicarboxyl­ate 1.67-hydrate

**DOI:** 10.1107/S1600536810027066

**Published:** 2010-07-14

**Authors:** Güneş Demirtaş, Necmi Dege, Okan Zafer Yeşilel, Hakan Erer, Orhan Büyükgüngör

**Affiliations:** a Department of Physics, Faculty of Arts and Sciences, Ondokuz Mayıs University, 55139 Samsun, Turkey; b Department of Chemistry, Faculty of Arts and Sciences, Osmangazi University, 26480 Eskişehir, Turkey

## Abstract

The asymmetric unit of the title compound, [Ni(C_2_H_8_N_2_)_3_](C_5_H_2_N_2_O_4_)·1.67H_2_O, consists of three [Ni(en)_3_]^2+^ dications (en is ethyl­enediamine), three [(pzdc)_3_]^2−^ dianions (pzdc is pyrazole-3,5-dicarboxyl­ate) and five water mol­ecules. In each complex dication, the Ni^II^ atom is coordinated by six N atoms from three en ligands forming a distorted octa­hedral coordination geometry. In the crystal, the ions and water mol­ecules are linked into a three-dimensional framework by a large number of N—H⋯O and O—H⋯O hydrogen bonds.

## Related literature

For the biological activity of pyrazole compounds, see: Chambers *et al.* (1985 ▶); Lee *et al.* (1989 ▶). For the crystal structures of pyrazole derivatives, see: Foces-Foces *et al.* (2006 ▶); Qu (2009 ▶); Xiao *et al.* (2007 ▶, 2009 ▶); Xiao & Zhao (2009 ▶); Yao *et al.* (2009 ▶). For Ni—N bond lengths, see: Emam *et al.* (2008 ▶).
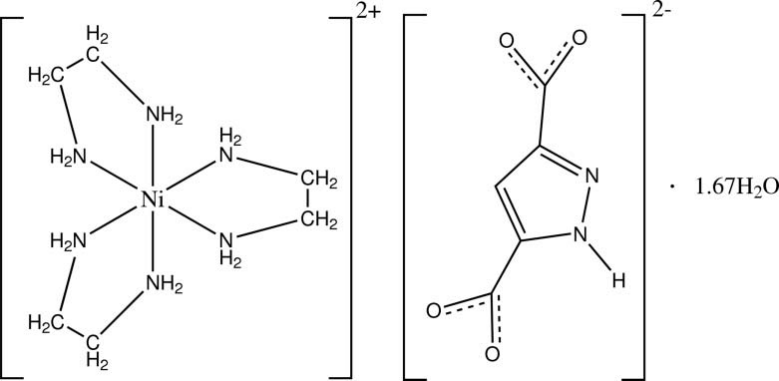


## Experimental

### 

#### Crystal data

[Ni(C_2_H_8_N_2_)_3_](C_5_H_2_N_2_O_4_)·1.67H_2_O
*M* *_r_* = 423.13
Triclinic, 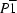

*a* = 12.8145 (7) Å
*b* = 12.9218 (7) Å
*c* = 18.6311 (13) Å
α = 72.008 (5)°
β = 75.446 (5)°
γ = 73.480 (4)°
*V* = 2767.5 (3) Å^3^
*Z* = 6
Mo *K*α radiation
μ = 1.10 mm^−1^
*T* = 296 K
0.52 × 0.35 × 0.07 mm

#### Data collection

Stoe IPDS 2 diffractometer
Absorption correction: integration (*X-RED32*; Stoe & Cie, 2002 ▶) *T*_min_ = 0.582, *T*_max_ = 0.949
42996 measured reflections
11465 independent reflections
6232 reflections with *I* > 2σ(*I*)
*R*_int_ = 0.091

#### Refinement

*R*[*F* ^2^ > 2σ(*F* ^2^)] = 0.058
*wR*(*F* ^2^) = 0.146
*S* = 0.91
11465 reflections
706 parameters
15 restraints
H atoms treated by a mixture of independent and constrained refinement
Δρ_max_ = 1.25 e Å^−3^
Δρ_min_ = −1.59 e Å^−3^

### 

Data collection: *X-AREA* (Stoe & Cie, 2002 ▶); cell refinement: *X-AREA*; data reduction: *X-RED32* (Stoe & Cie, 2002 ▶); program(s) used to solve structure: *SHELXS97* (Sheldrick, 2008 ▶); program(s) used to refine structure: *SHELXL97* (Sheldrick, 2008 ▶); molecular graphics: *ORTEP-3 for Windows* (Farrugia, 1997 ▶); software used to prepare material for publication: *WinGX* (Farrugia, 1999 ▶) and *PLATON* (Spek, 2009 ▶).

## Supplementary Material

Crystal structure: contains datablocks I, global. DOI: 10.1107/S1600536810027066/ci5127sup1.cif

Structure factors: contains datablocks I. DOI: 10.1107/S1600536810027066/ci5127Isup2.hkl

Additional supplementary materials:  crystallographic information; 3D view; checkCIF report

## Figures and Tables

**Table 1:** Hydrogen-bond geometry (Å, °)

*D*—H⋯*A*	*D*—H	H⋯*A*	*D*⋯*A*	*D*—H⋯*A*
O1*W*—H1*W*1⋯O4	0.82 (5)	1.93 (3)	2.725 (6)	162 (8)
O1*W*—H2*W*1⋯O9	0.83 (8)	1.94 (8)	2.739 (8)	163 (8)
O2*W*—H1*W*2⋯O6	0.82 (7)	1.90 (3)	2.674 (5)	159 (6)
O2*W*—H2*W*2⋯O1	0.83 (6)	2.04 (3)	2.856 (6)	167 (8)
O3*W*—H1*W*3⋯O7	0.84 (6)	1.95 (6)	2.787 (6)	175 (2)
O3*W*—H2*W*3⋯O11^i^	0.84 (6)	2.36 (4)	3.074 (6)	144 (6)
O3*W*—H2*W*3⋯N24^i^	0.84 (6)	2.55 (5)	3.236 (6)	141 (7)
O4*W*—H1*W*4⋯O1*W*^ii^	0.83 (8)	2.37 (5)	3.094 (8)	146 (8)
O4*W*—H1*W*4⋯O9^ii^	0.83 (8)	2.57 (5)	3.293 (6)	146 (8)
O4*W*—H2*W*4⋯O5*W*	0.84 (7)	2.03 (7)	2.839 (6)	163 (7)
O5*W*—H1*W*5⋯O12^iii^	0.83 (2)	2.01 (3)	2.819 (5)	165 (6)
O5*W*—H2*W*5⋯O12^ii^	0.84 (4)	2.06 (2)	2.888 (5)	176 (7)
N1—H1*C*⋯O1^ii^	0.90	2.34	3.215 (6)	164
N1—H1*D*⋯O3*W*^ii^	0.90	2.27	3.159 (7)	170
N2—H2*C*⋯O9^iv^	0.90	2.25	3.118 (6)	162
N2—H2*D*⋯O1^iv^	0.90	2.55	3.358 (6)	150
N3—H3*D*⋯N21^v^	0.90	2.24	3.066 (5)	153
N4—H4*C*⋯O2^ii^	0.90	2.24	3.091 (5)	158
N4—H4*D*⋯O1^iv^	0.90	2.15	3.039 (5)	168
N5—H5*C*⋯O2^ii^	0.90	2.28	3.091 (6)	150
N5—H5*D*⋯O5^v^	0.90	2.02	2.913 (5)	174
N6—H6*C*⋯O10^iv^	0.90	2.38	3.154 (6)	144
N6—H6*C*⋯O9^iv^	0.90	2.54	3.357 (6)	152
N7—H7*C*⋯N19^vi^	0.90	2.07	2.950 (5)	165
N7—H7*D*⋯O3*W*	0.90	2.45	3.314 (6)	162
N8—H8*C*⋯O2*W*	0.90	2.22	3.029 (6)	149
N8—H8*D*⋯O5^vii^	0.90	2.57	3.388 (6)	152
N8—H8*D*⋯O6^vii^	0.90	2.59	3.345 (5)	142
N9—H9*C*⋯O2^vi^	0.90	2.19	3.071 (5)	166
N9—H9*D*⋯O5^vii^	0.90	2.18	3.049 (6)	162
N10—H10*D*⋯O11^i^	0.90	2.21	3.066 (6)	159
N11—H11*C*⋯O1*W*^vii^	0.90	2.33	3.173 (7)	156
N11—H11*D*⋯O6^vii^	0.90	2.09	2.959 (6)	162
N12—H12*C*⋯O2*W*	0.90	2.22	3.100 (7)	166
N12—H12*D*⋯O3*W*	0.90	2.46	3.213 (6)	142
N13—H13*C*⋯O8^viii^	0.90	2.08	2.929 (6)	157
N13—H13*D*⋯O7	0.90	2.57	3.307 (7)	139
N14—H14*C*⋯O4^vii^	0.90	2.08	2.976 (6)	173
N14—H14*D*⋯O12^vii^	0.90	2.41	3.284 (7)	163
N15—H15*C*⋯O3^vii^	0.90	2.13	2.940 (6)	149
N16—H16*C*⋯O8^viii^	0.90	2.20	3.017 (6)	151
N16—H16*D*⋯O12^vii^	0.90	2.27	3.147 (5)	164
N17—H17*C*⋯O7^viii^	0.90	2.42	3.290 (6)	162
N17—H17*C*⋯O8^viii^	0.90	2.59	3.351 (6)	142
N17—H17*D*⋯O10^ix^	0.90	2.31	3.151 (6)	155
N18—H18*C*⋯O3^vii^	0.90	2.25	3.100 (6)	157
N20—H20⋯O11^x^	0.86	2.35	3.058 (6)	140
N22—H22⋯O4*W*	0.86	2.00	2.819 (5)	160
N23—H23⋯O10^xi^	0.86	2.03	2.856 (6)	160

Symmetry codes: (i) 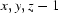; (ii) 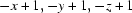; (iii) 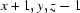; (iv) 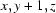; (v) 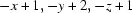; (vi) 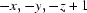; (vii) 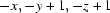; (viii) 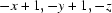; (ix) 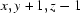; (x) 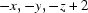; (xi) 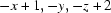.

**Fig. 1.**
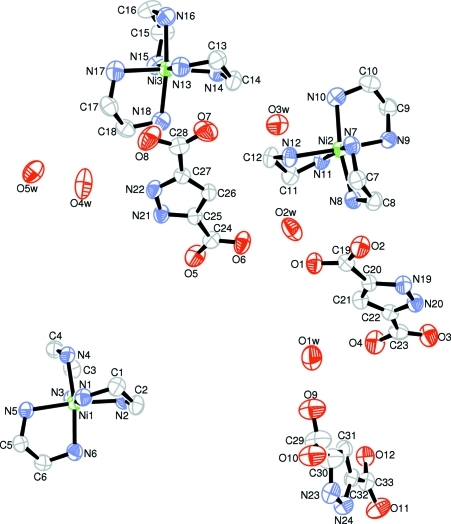
The asymmetric unit of the title compound, showing the atomic numbering scheme. Displacement ellipsoids are drawn at the 50% probability level.

## supplementary crystallographic information

### Comment

Pyrazole derivatives exhibit biological activities (Lee *et al.*, 1989; Chambers *et al.*,1985). As a result, crystal structures of many pyrazole derivatives have been reported (Xiao *et al.*, 2007, 2009; Xiao & Zhao, 2009; Foces-Foces *et al.*, 2006; Qu *et al.*, 2009; Yao *et al.*, 2009). We report here the crystal structure of the title compound, {[Ni(en)_3_](pzdc)}_3_.5H_2_O.

The asymmetric unit of the title compound consists of three [Ni(en)_3_]^2+^ cations, three [(pzdc)_3_]^2-^ dianions and five water molecules (Fig. 1). In each complex cation, the Ni^II^ ion is six-coordinated by six N atoms from three pzdc ligands in a distorted octahedral coordination geometry. The Ni—N bond distances range from 2.108 (5) Å to 2.130 (4) Å for Ni1, from 2.106 (4) Å to 2.130 (4) Å for Ni2, and from 2.121 (5) Å to 2.147 (5) Å for Ni3 atom. These values are consistent with those reported in the literature (Emam *et al.*, 2008). The N—Ni—N bond angles range from 81.46 (18)° to 172.19 (18)° for Ni1, from 81.63 (16)° to 175.39 (16)° for Ni2, and from 81.65 (19)° to 172.13 (18)° for Ni3 atom.

In the crystal structure, the ions and water molecules are linked through a large number of N—H···O and O—H···O hydrogen bonds (Table 1) into a three-dimensional framework.

### Experimental

A solution of 3,5-pyrazoledicarboxylic acid monohydrate (0.5 g, 2.87 mmol) in water (20 ml) was added dropwise with stirring at 323 K to a solution of nickel(II) acetatetetrahydrate (0.71 g, 2.87 mmol) in distilled water (15 ml). The solution immediately became a suspension and was stirred for 2 h at 323 K. Then ethylenediamine (0.51 g, 8.61 mmol) in water (10 ml) was added dropwise to this suspension. The clear solution obtained was stirred for 2 h at 323 K and then cooled to room temperature. Single crystals formed were filtered and washed with 10 ml of water and were dried in air.

### Refinement

H atoms of the water molecules were located in a difference map and were refined with O–H and H···H distance restraints of 0.83 (1) and 1.35 (1) Å, respectively. H atoms attached to C and N atoms were positioned geometrically [N–H = 0.86 or 0.90 Å and C–H = 0.93 or 0.97 Å] and treated as riding with *U*_iso_(H) = 1.1Ueq (C,N).

### Crystal data

**Table:**

[Ni(C_2_H_8_N_2_)_3_](C_5_H_2_N_2_O_4_)·1.67H_2_O	*Z* = 6
*M**_r_* = 423.13	*F*(000) = 1348
Triclinic, *P*1	*D*_x_ = 1.523 Mg m^−^^3^
Hall symbol: -P 1	Mo *K*α radiation, λ = 0.71073 Å
*a* = 12.8145 (7) Å	Cell parameters from 64545 reflections
*b* = 12.9218 (7) Å	θ = 1.7–27.2°
*c* = 18.6311 (13) Å	µ = 1.10 mm^−^^1^
α = 72.008 (5)°	*T* = 296 K
β = 75.446 (5)°	Prism, violet
γ = 73.480 (4)°	0.52 × 0.35 × 0.07 mm
*V* = 2767.5 (3) Å^3^	

### Data collection

**Table:**

Stoe IPDS 2 diffractometer	11465 independent reflections
Radiation source: fine-focus sealed tube	6232 reflections with *I* > 2σ(*I*)
graphite	*R*_int_ = 0.091
w–scan rotation	θ_max_ = 26.5°, θ_min_ = 1.7°
Absorption correction: integration (*X-RED32*; Stoe & Cie, 2002)	*h* = −16→16
*T*_min_ = 0.582, *T*_max_ = 0.949	*k* = −16→16
42996 measured reflections	*l* = −23→23

### Refinement

**Table:**

Refinement on *F*^2^	Primary atom site location: structure-invariant direct methods
Least-squares matrix: full	Secondary atom site location: difference Fourier map
*R*[*F*^2^ > 2σ(*F*^2^)] = 0.058	Hydrogen site location: inferred from neighbouring sites
*wR*(*F*^2^) = 0.146	H atoms treated by a mixture of independent and constrained refinement
*S* = 0.91	*w* = 1/[σ^2^(*F*_o_^2^) + (0.0702*P*)^2^] where *P* = (*F*_o_^2^ + 2*F*_c_^2^)/3
11465 reflections	(Δ/σ)_max_ = 0.001
706 parameters	Δρ_max_ = 1.25 e Å^−^^3^
15 restraints	Δρ_min_ = −1.59 e Å^−^^3^

### Special details

**Table:**

Geometry. All e.s.d.'s (except the e.s.d. in the dihedral angle between two l.s. planes) are estimated using the full covariance matrix. The cell e.s.d.'s are taken into account individually in the estimation of e.s.d.'s in distances, angles and torsion angles; correlations between e.s.d.'s in cell parameters are only used when they are defined by crystal symmetry. An approximate (isotropic) treatment of cell e.s.d.'s is used for estimating e.s.d.'s involving l.s. planes.
Refinement. Refinement of *F*^2^ against ALL reflections. The weighted *R*-factor *wR* and goodness of fit *S* are based on *F*^2^, conventional *R*-factors *R* are based on *F*, with *F* set to zero for negative *F*^2^. The threshold expression of *F*^2^ > σ(*F*^2^) is used only for calculating *R*-factors(gt) *etc*. and is not relevant to the choice of reflections for refinement. *R*-factors based on *F*^2^ are statistically about twice as large as those based on *F*, and *R*- factors based on ALL data will be even larger.

### Fractional atomic coordinates and isotropic or equivalent isotropic displacement parameters (Å^2^)

**Table:**

	*x*	*y*	*z*	*U*_iso_*/*U*_eq_	
C1	0.5179 (5)	0.8673 (4)	0.6477 (3)	0.0582 (15)	
H1A	0.5433	0.7870	0.6561	0.070*	
H1B	0.4812	0.8958	0.6036	0.070*	
C2	0.4380 (5)	0.8948 (4)	0.7174 (4)	0.0583 (15)	
H2A	0.3731	0.8652	0.7260	0.070*	
H2B	0.4727	0.8617	0.7624	0.070*	
C3	0.4048 (5)	1.2976 (5)	0.5923 (4)	0.0623 (16)	
H3A	0.3873	1.3782	0.5837	0.075*	
H3B	0.3360	1.2739	0.6021	0.075*	
C4	0.4784 (5)	1.2657 (5)	0.5223 (4)	0.0604 (15)	
H4A	0.4393	1.2947	0.4793	0.073*	
H4B	0.5437	1.2963	0.5087	0.073*	
C5	0.7686 (4)	1.0853 (5)	0.6721 (3)	0.0574 (15)	
H5A	0.8242	1.1282	0.6614	0.069*	
H5B	0.8059	1.0077	0.6756	0.069*	
C6	0.6919 (4)	1.0963 (5)	0.7464 (3)	0.0573 (14)	
H6A	0.7323	1.0635	0.7886	0.069*	
H6B	0.6604	1.1745	0.7449	0.069*	
N1	0.6134 (3)	0.9188 (3)	0.6332 (3)	0.0513 (11)	
H1C	0.6499	0.9231	0.5847	0.062*	
H1D	0.6606	0.8775	0.6654	0.062*	
N2	0.4062 (3)	1.0173 (3)	0.7039 (3)	0.0523 (11)	
H2C	0.3767	1.0359	0.7487	0.063*	
H2D	0.3553	1.0461	0.6732	0.063*	
N3	0.4596 (3)	1.2453 (3)	0.6588 (3)	0.0543 (12)	
H3C	0.4091	1.2420	0.7023	0.065*	
H3D	0.5062	1.2857	0.6591	0.065*	
N4	0.5103 (3)	1.1424 (3)	0.5405 (3)	0.0499 (11)	
H4C	0.5694	1.1204	0.5062	0.060*	
H4D	0.4542	1.1152	0.5383	0.060*	
N5	0.7031 (3)	1.1269 (3)	0.6100 (3)	0.0474 (10)	
H5C	0.7399	1.0975	0.5703	0.057*	
H5D	0.6925	1.2017	0.5934	0.057*	
N6	0.6034 (3)	1.0385 (4)	0.7576 (3)	0.0546 (12)	
H6C	0.5470	1.0595	0.7939	0.065*	
H6D	0.6290	0.9641	0.7729	0.065*	
Ni1	0.54902 (5)	1.08143 (5)	0.65171 (4)	0.04029 (17)	
C7	0.0582 (4)	0.1370 (4)	0.4329 (3)	0.0499 (13)	
H7A	0.1290	0.1529	0.4299	0.060*	
H7B	0.0690	0.0572	0.4407	0.060*	
C8	−0.0235 (4)	0.1734 (4)	0.4997 (3)	0.0521 (13)	
H8A	−0.0918	0.1503	0.5065	0.063*	
H8B	0.0068	0.1393	0.5465	0.063*	
C9	−0.2880 (4)	0.3775 (5)	0.3375 (3)	0.0557 (14)	
H9A	−0.3128	0.4569	0.3336	0.067*	
H9B	−0.3524	0.3473	0.3456	0.067*	
C10	−0.2115 (5)	0.3580 (5)	0.2650 (4)	0.0601 (15)	
H10A	−0.1920	0.2790	0.2670	0.072*	
H10B	−0.2479	0.3980	0.2213	0.072*	
C11	−0.0287 (4)	0.5638 (4)	0.3781 (3)	0.0556 (14)	
H11A	−0.0032	0.5307	0.4267	0.067*	
H11B	−0.0472	0.6443	0.3696	0.067*	
C12	0.0612 (4)	0.5309 (4)	0.3144 (3)	0.0540 (14)	
H12A	0.0377	0.5672	0.2653	0.065*	
H12B	0.1276	0.5534	0.3136	0.065*	
N7	0.0171 (3)	0.1960 (3)	0.3615 (3)	0.0473 (11)	
H7C	−0.0282	0.1595	0.3543	0.057*	
H7D	0.0741	0.1986	0.3218	0.057*	
N8	−0.0452 (3)	0.2964 (3)	0.4829 (3)	0.0485 (11)	
H8C	0.0127	0.3174	0.4889	0.058*	
H8D	−0.1059	0.3225	0.5146	0.058*	
N9	−0.2290 (3)	0.3226 (3)	0.4019 (2)	0.0465 (10)	
H9C	−0.2230	0.2484	0.4143	0.056*	
H9D	−0.2658	0.3483	0.4431	0.056*	
N10	−0.1108 (3)	0.3976 (4)	0.2566 (3)	0.0548 (11)	
H10C	−0.1229	0.4719	0.2357	0.066*	
H10D	−0.0547	0.3640	0.2255	0.066*	
N11	−0.1269 (3)	0.5248 (3)	0.3801 (3)	0.0494 (11)	
H11C	−0.1627	0.5692	0.3418	0.059*	
H11D	−0.1736	0.5257	0.4251	0.059*	
N12	0.0836 (3)	0.4093 (3)	0.3281 (3)	0.0491 (11)	
H12C	0.1272	0.3759	0.3637	0.059*	
H12D	0.1188	0.3892	0.2845	0.059*	
Ni2	−0.06954 (5)	0.35919 (5)	0.36689 (4)	0.03914 (17)	
C13	0.2460 (5)	0.4831 (4)	0.0518 (3)	0.0571 (15)	
H13A	0.2734	0.4037	0.0720	0.069*	
H13B	0.2123	0.4937	0.0082	0.069*	
C14	0.1618 (4)	0.5309 (4)	0.1126 (3)	0.0565 (15)	
H14A	0.0991	0.4961	0.1279	0.068*	
H14B	0.1943	0.5170	0.1574	0.068*	
C15	0.1245 (5)	0.9129 (5)	−0.0625 (4)	0.0708 (17)	
H15A	0.1061	0.9934	−0.0827	0.085*	
H15B	0.0564	0.8866	−0.0451	0.085*	
C16	0.1991 (6)	0.8584 (5)	−0.1240 (4)	0.0714 (17)	
H16A	0.1615	0.8743	−0.1666	0.086*	
H16B	0.2658	0.8872	−0.1430	0.086*	
C17	0.4890 (4)	0.7190 (5)	0.0333 (4)	0.0573 (15)	
H17A	0.5539	0.7505	0.0167	0.069*	
H17B	0.5128	0.6385	0.0506	0.069*	
C18	0.4118 (4)	0.7649 (5)	0.0977 (4)	0.0575 (15)	
H18A	0.4490	0.7450	0.1410	0.069*	
H18B	0.3911	0.8457	0.0808	0.069*	
N13	0.3379 (4)	0.5407 (3)	0.0279 (3)	0.0564 (12)	
H13C	0.3777	0.5303	−0.0176	0.068*	
H13D	0.3828	0.5135	0.0628	0.068*	
N14	0.1256 (4)	0.6523 (4)	0.0802 (3)	0.0574 (12)	
H14C	0.0917	0.6864	0.1181	0.069*	
H14D	0.0777	0.6656	0.0487	0.069*	
N15	0.1838 (4)	0.8835 (4)	0.0012 (3)	0.0621 (13)	
H15C	0.1357	0.8944	0.0440	0.075*	
H15D	0.2324	0.9270	−0.0097	0.075*	
N16	0.2278 (4)	0.7364 (4)	−0.0897 (3)	0.0586 (12)	
H16C	0.2857	0.7035	−0.1201	0.070*	
H16D	0.1699	0.7063	−0.0839	0.070*	
N17	0.4282 (4)	0.7491 (4)	−0.0312 (3)	0.0592 (13)	
H17C	0.4643	0.7086	−0.0652	0.071*	
H17D	0.4217	0.8220	−0.0555	0.071*	
N18	0.3118 (4)	0.7189 (4)	0.1212 (3)	0.0594 (12)	
H18C	0.2560	0.7627	0.1461	0.071*	
H18D	0.3254	0.6499	0.1528	0.071*	
Ni3	0.26857 (5)	0.71329 (5)	0.01860 (4)	0.04165 (18)	
C19	0.2427 (4)	0.0035 (4)	0.5734 (3)	0.0423 (12)	
C20	0.1570 (4)	0.0170 (4)	0.6440 (3)	0.0383 (11)	
C21	0.1256 (4)	0.1049 (4)	0.6794 (3)	0.0438 (12)	
H21	0.1559	0.1673	0.6652	0.053*	
C22	0.0413 (4)	0.0806 (4)	0.7392 (3)	0.0410 (11)	
C23	−0.0293 (4)	0.1371 (4)	0.7996 (3)	0.0498 (13)	
N19	0.0961 (3)	−0.0585 (3)	0.6795 (2)	0.0448 (10)	
N20	0.0263 (3)	−0.0180 (3)	0.7365 (2)	0.0463 (10)	
H20	−0.0230	−0.0511	0.7682	0.056*	
O1	0.2991 (3)	0.0760 (3)	0.5451 (2)	0.0532 (9)	
O2	0.2527 (3)	−0.0794 (3)	0.5490 (2)	0.0597 (10)	
O3	−0.1076 (3)	0.0967 (3)	0.8401 (2)	0.0678 (12)	
O4	−0.0034 (3)	0.2208 (3)	0.8036 (2)	0.0678 (12)	
C24	0.3028 (4)	0.5553 (4)	0.4199 (3)	0.0475 (13)	
C25	0.3711 (4)	0.5281 (4)	0.3483 (3)	0.0405 (11)	
C26	0.3625 (4)	0.4521 (4)	0.3106 (3)	0.0424 (12)	
H26	0.3120	0.4066	0.3262	0.051*	
C27	0.4437 (4)	0.4588 (4)	0.2468 (3)	0.0410 (11)	
C28	0.4774 (4)	0.4047 (4)	0.1820 (3)	0.0508 (13)	
N21	0.4528 (3)	0.5795 (3)	0.3084 (3)	0.0476 (11)	
N22	0.4953 (3)	0.5362 (3)	0.2476 (2)	0.0479 (11)	
H22	0.5496	0.5557	0.2128	0.057*	
O5	0.3164 (4)	0.6324 (3)	0.4400 (2)	0.0716 (12)	
O6	0.2352 (3)	0.4949 (3)	0.4575 (2)	0.0657 (12)	
O7	0.4196 (4)	0.3450 (4)	0.1783 (3)	0.0773 (13)	
O8	0.5616 (4)	0.4255 (4)	0.1348 (3)	0.0832 (14)	
C29	0.3700 (5)	0.0707 (6)	0.9111 (4)	0.0794 (8)	
C30	0.2975 (5)	0.1155 (6)	0.9743 (4)	0.0794 (8)	
C31	0.1929 (4)	0.1807 (4)	0.9841 (3)	0.0474 (13)	
H31	0.1464	0.2116	0.9481	0.057*	
C32	0.1700 (4)	0.1916 (4)	1.0591 (3)	0.0450 (12)	
C33	0.0673 (4)	0.2544 (4)	1.1000 (3)	0.0496 (13)	
N23	0.3316 (3)	0.0913 (3)	1.0412 (3)	0.0537 (12)	
H23	0.3956	0.0506	1.0493	0.064*	
N24	0.2560 (4)	0.1369 (4)	1.0951 (3)	0.0526 (11)	
O9	0.3421 (3)	0.1163 (4)	0.8460 (3)	0.0794 (8)	
O10	0.4521 (3)	−0.0015 (4)	0.9213 (3)	0.0794 (8)	
O11	0.0524 (4)	0.2313 (3)	1.1713 (2)	0.0737 (12)	
O12	0.0028 (3)	0.3259 (3)	1.0575 (2)	0.0560 (10)	
O1W	0.1964 (5)	0.2779 (5)	0.7625 (3)	0.0983 (16)	
H1W1	0.132 (3)	0.273 (7)	0.768 (5)	0.147*	
H2W1	0.229 (6)	0.225 (6)	0.793 (5)	0.147*	
O2W	0.1946 (4)	0.2970 (3)	0.4715 (3)	0.0768 (13)	
H1W2	0.221 (6)	0.347 (4)	0.473 (4)	0.115*	
H2W2	0.223 (6)	0.237 (3)	0.499 (4)	0.115*	
O3W	0.2482 (4)	0.2325 (4)	0.2382 (3)	0.0741 (12)	
H1W3	0.301 (4)	0.264 (6)	0.218 (3)	0.111*	
H2W3	0.219 (5)	0.231 (6)	0.203 (3)	0.111*	
O4W	0.6821 (4)	0.6201 (4)	0.1617 (3)	0.0881 (16)	
H1W4	0.698 (6)	0.672 (5)	0.171 (5)	0.132*	
H2W4	0.737 (4)	0.590 (6)	0.134 (4)	0.132*	
O5W	0.8951 (3)	0.5382 (3)	0.0821 (2)	0.0658 (11)	
H1W5	0.918 (5)	0.4715 (19)	0.081 (4)	0.099*	
H2W5	0.924 (5)	0.576 (4)	0.040 (2)	0.099*	

### Atomic displacement parameters (Å^2^)

**Table:**

	*U*^11^	*U*^22^	*U*^33^	*U*^12^	*U*^13^	*U*^23^
C1	0.068 (4)	0.048 (3)	0.061 (4)	−0.016 (3)	−0.004 (3)	−0.021 (3)
C2	0.061 (3)	0.051 (3)	0.063 (4)	−0.021 (3)	0.000 (3)	−0.015 (3)
C3	0.051 (3)	0.050 (3)	0.084 (5)	−0.003 (3)	−0.011 (3)	−0.022 (3)
C4	0.055 (3)	0.058 (3)	0.063 (4)	−0.010 (3)	−0.017 (3)	−0.006 (3)
C5	0.042 (3)	0.072 (4)	0.056 (4)	−0.016 (3)	−0.006 (3)	−0.013 (3)
C6	0.059 (3)	0.066 (4)	0.051 (4)	−0.016 (3)	−0.012 (3)	−0.018 (3)
N1	0.051 (2)	0.052 (3)	0.051 (3)	−0.011 (2)	−0.003 (2)	−0.019 (2)
N2	0.048 (2)	0.057 (3)	0.057 (3)	−0.015 (2)	−0.003 (2)	−0.022 (2)
N3	0.047 (2)	0.053 (3)	0.069 (3)	−0.017 (2)	0.000 (2)	−0.026 (2)
N4	0.047 (2)	0.051 (3)	0.051 (3)	−0.013 (2)	−0.005 (2)	−0.014 (2)
N5	0.042 (2)	0.050 (2)	0.049 (3)	−0.011 (2)	−0.003 (2)	−0.014 (2)
N6	0.045 (2)	0.065 (3)	0.054 (3)	−0.015 (2)	−0.002 (2)	−0.020 (2)
Ni1	0.0378 (3)	0.0423 (3)	0.0431 (4)	−0.0097 (3)	−0.0033 (3)	−0.0166 (3)
C7	0.048 (3)	0.037 (3)	0.064 (4)	−0.008 (2)	−0.012 (3)	−0.012 (3)
C8	0.050 (3)	0.046 (3)	0.053 (4)	−0.012 (3)	−0.009 (3)	−0.002 (3)
C9	0.043 (3)	0.059 (3)	0.063 (4)	−0.006 (3)	−0.013 (3)	−0.015 (3)
C10	0.064 (4)	0.058 (3)	0.062 (4)	−0.003 (3)	−0.021 (3)	−0.023 (3)
C11	0.062 (3)	0.044 (3)	0.061 (4)	−0.012 (3)	−0.006 (3)	−0.017 (3)
C12	0.055 (3)	0.042 (3)	0.059 (4)	−0.018 (3)	0.003 (3)	−0.008 (3)
N7	0.045 (2)	0.043 (2)	0.057 (3)	−0.0115 (19)	−0.005 (2)	−0.019 (2)
N8	0.044 (2)	0.046 (2)	0.056 (3)	−0.011 (2)	−0.006 (2)	−0.017 (2)
N9	0.043 (2)	0.047 (2)	0.046 (3)	−0.0086 (19)	−0.005 (2)	−0.012 (2)
N10	0.048 (2)	0.054 (3)	0.054 (3)	−0.003 (2)	−0.007 (2)	−0.011 (2)
N11	0.045 (2)	0.042 (2)	0.054 (3)	−0.0074 (19)	0.004 (2)	−0.014 (2)
N12	0.040 (2)	0.042 (2)	0.059 (3)	−0.0080 (19)	−0.002 (2)	−0.011 (2)
Ni2	0.0380 (3)	0.0353 (3)	0.0421 (4)	−0.0070 (3)	−0.0037 (3)	−0.0110 (3)
C13	0.071 (4)	0.044 (3)	0.057 (4)	−0.017 (3)	−0.010 (3)	−0.013 (3)
C14	0.058 (3)	0.054 (3)	0.055 (4)	−0.021 (3)	0.003 (3)	−0.013 (3)
C15	0.079 (4)	0.055 (4)	0.070 (5)	0.009 (3)	−0.026 (4)	−0.017 (3)
C16	0.088 (5)	0.070 (4)	0.043 (4)	−0.003 (4)	−0.014 (3)	−0.008 (3)
C17	0.050 (3)	0.053 (3)	0.074 (4)	−0.010 (3)	−0.011 (3)	−0.025 (3)
C18	0.062 (3)	0.054 (3)	0.066 (4)	−0.010 (3)	−0.016 (3)	−0.027 (3)
N13	0.057 (3)	0.054 (3)	0.059 (3)	−0.009 (2)	−0.002 (2)	−0.024 (2)
N14	0.059 (3)	0.056 (3)	0.057 (3)	−0.011 (2)	−0.005 (2)	−0.020 (2)
N15	0.065 (3)	0.054 (3)	0.060 (3)	−0.004 (2)	−0.006 (3)	−0.018 (2)
N16	0.055 (3)	0.063 (3)	0.058 (3)	−0.011 (2)	−0.004 (2)	−0.024 (3)
N17	0.057 (3)	0.053 (3)	0.065 (3)	−0.010 (2)	0.002 (2)	−0.025 (2)
N18	0.061 (3)	0.057 (3)	0.057 (3)	−0.008 (2)	0.000 (2)	−0.022 (2)
Ni3	0.0409 (3)	0.0397 (3)	0.0433 (4)	−0.0069 (3)	−0.0033 (3)	−0.0145 (3)
C19	0.039 (3)	0.043 (3)	0.047 (3)	−0.006 (2)	−0.007 (2)	−0.016 (2)
C20	0.036 (2)	0.037 (2)	0.042 (3)	−0.009 (2)	−0.006 (2)	−0.010 (2)
C21	0.041 (3)	0.038 (3)	0.052 (3)	−0.007 (2)	−0.005 (2)	−0.016 (2)
C22	0.042 (3)	0.037 (3)	0.045 (3)	−0.004 (2)	−0.010 (2)	−0.015 (2)
C23	0.049 (3)	0.055 (3)	0.047 (3)	0.000 (3)	−0.012 (3)	−0.023 (3)
N19	0.044 (2)	0.043 (2)	0.049 (3)	−0.0107 (19)	0.000 (2)	−0.019 (2)
N20	0.042 (2)	0.051 (2)	0.048 (3)	−0.014 (2)	0.001 (2)	−0.020 (2)
O1	0.0483 (19)	0.054 (2)	0.059 (3)	−0.0201 (17)	0.0051 (17)	−0.0202 (18)
O2	0.073 (2)	0.053 (2)	0.057 (3)	−0.0213 (19)	0.010 (2)	−0.0291 (19)
O3	0.054 (2)	0.086 (3)	0.069 (3)	−0.025 (2)	0.016 (2)	−0.043 (2)
O4	0.075 (3)	0.054 (2)	0.078 (3)	−0.016 (2)	0.010 (2)	−0.038 (2)
C24	0.055 (3)	0.040 (3)	0.046 (3)	−0.007 (2)	−0.003 (3)	−0.016 (2)
C25	0.042 (3)	0.031 (2)	0.049 (3)	−0.008 (2)	−0.006 (2)	−0.012 (2)
C26	0.040 (2)	0.042 (3)	0.047 (3)	−0.013 (2)	−0.002 (2)	−0.015 (2)
C27	0.043 (3)	0.036 (2)	0.045 (3)	−0.005 (2)	−0.008 (2)	−0.015 (2)
C28	0.057 (3)	0.044 (3)	0.048 (3)	−0.001 (3)	−0.011 (3)	−0.015 (3)
N21	0.049 (2)	0.045 (2)	0.050 (3)	−0.016 (2)	0.001 (2)	−0.017 (2)
N22	0.046 (2)	0.051 (2)	0.046 (3)	−0.016 (2)	0.005 (2)	−0.017 (2)
O5	0.107 (3)	0.054 (2)	0.062 (3)	−0.032 (2)	0.010 (2)	−0.034 (2)
O6	0.074 (3)	0.060 (2)	0.064 (3)	−0.031 (2)	0.021 (2)	−0.029 (2)
O7	0.088 (3)	0.084 (3)	0.078 (3)	−0.021 (3)	−0.014 (3)	−0.046 (3)
O8	0.095 (3)	0.084 (3)	0.062 (3)	−0.022 (3)	0.023 (3)	−0.035 (2)
C29	0.0634 (15)	0.099 (2)	0.0613 (18)	0.0140 (14)	−0.0077 (14)	−0.0332 (16)
C30	0.0634 (15)	0.099 (2)	0.0613 (18)	0.0140 (14)	−0.0077 (14)	−0.0332 (16)
C31	0.041 (3)	0.053 (3)	0.049 (3)	−0.011 (2)	−0.005 (2)	−0.016 (3)
C32	0.043 (3)	0.040 (3)	0.046 (3)	−0.010 (2)	0.005 (2)	−0.013 (2)
C33	0.053 (3)	0.043 (3)	0.053 (4)	−0.012 (3)	0.002 (3)	−0.021 (3)
N23	0.044 (2)	0.041 (2)	0.063 (3)	0.0027 (19)	0.003 (2)	−0.015 (2)
N24	0.051 (3)	0.052 (3)	0.050 (3)	−0.003 (2)	−0.003 (2)	−0.019 (2)
O9	0.0634 (15)	0.099 (2)	0.0613 (18)	0.0140 (14)	−0.0077 (14)	−0.0332 (16)
O10	0.0634 (15)	0.099 (2)	0.0613 (18)	0.0140 (14)	−0.0077 (14)	−0.0332 (16)
O11	0.084 (3)	0.071 (3)	0.050 (3)	0.004 (2)	0.004 (2)	−0.024 (2)
O12	0.047 (2)	0.052 (2)	0.066 (3)	−0.0041 (18)	−0.0037 (19)	−0.021 (2)
O1W	0.107 (4)	0.107 (4)	0.093 (4)	−0.056 (4)	−0.006 (3)	−0.022 (3)
O2W	0.079 (3)	0.056 (2)	0.110 (4)	−0.024 (2)	−0.042 (3)	−0.014 (3)
O3W	0.074 (3)	0.085 (3)	0.065 (3)	−0.021 (2)	−0.016 (2)	−0.015 (3)
O4W	0.072 (3)	0.065 (3)	0.115 (4)	−0.030 (2)	0.029 (3)	−0.029 (3)
O5W	0.071 (3)	0.056 (2)	0.057 (3)	−0.008 (2)	0.004 (2)	−0.014 (2)

### Geometric parameters (Å, °)

**Table:**

C1—N1	1.483 (6)	C14—H14A	0.97
C1—C2	1.507 (7)	C14—H14B	0.97
C1—H1A	0.97	C15—N15	1.462 (8)
C1—H1B	0.97	C15—C16	1.512 (8)
C2—N2	1.474 (6)	C15—H15A	0.97
C2—H2A	0.97	C15—H15B	0.97
C2—H2B	0.97	C16—N16	1.482 (7)
C3—N3	1.461 (7)	C16—H16A	0.97
C3—C4	1.502 (8)	C16—H16B	0.97
C3—H3A	0.97	C17—N17	1.486 (7)
C3—H3B	0.97	C17—C18	1.510 (8)
C4—N4	1.478 (7)	C17—H17A	0.97
C4—H4A	0.97	C17—H17B	0.97
C4—H4B	0.97	C18—N18	1.476 (6)
C5—N5	1.475 (7)	C18—H18A	0.97
C5—C6	1.505 (7)	C18—H18B	0.97
C5—H5A	0.97	N13—Ni3	2.122 (4)
C5—H5B	0.97	N13—H13C	0.90
C6—N6	1.467 (6)	N13—H13D	0.90
C6—H6A	0.97	N14—Ni3	2.122 (4)
C6—H6B	0.97	N14—H14C	0.90
N1—Ni1	2.130 (4)	N14—H14D	0.90
N1—H1C	0.90	N15—Ni3	2.121 (5)
N1—H1D	0.90	N15—H15C	0.90
N2—Ni1	2.110 (4)	N15—H15D	0.90
N2—H2C	0.90	N16—Ni3	2.122 (5)
N2—H2D	0.90	N16—H16C	0.90
N3—Ni1	2.128 (4)	N16—H16D	0.90
N3—H3C	0.90	N17—Ni3	2.140 (4)
N3—H3D	0.90	N17—H17C	0.90
N4—Ni1	2.108 (5)	N17—H17D	0.90
N4—H4C	0.90	N18—Ni3	2.147 (5)
N4—H4D	0.90	N18—H18C	0.90
N5—Ni1	2.111 (3)	N18—H18D	0.90
N5—H5C	0.90	C19—O1	1.251 (5)
N5—H5D	0.90	C19—O2	1.252 (5)
N6—Ni1	2.118 (5)	C19—C20	1.507 (6)
N6—H6C	0.90	C20—N19	1.331 (5)
N6—H6D	0.90	C20—C21	1.399 (6)
C7—N7	1.457 (7)	C21—C22	1.371 (6)
C7—C8	1.514 (7)	C21—H21	0.93
C7—H7A	0.97	C22—N20	1.358 (5)
C7—H7B	0.97	C22—C23	1.496 (7)
C8—N8	1.479 (6)	C23—O3	1.234 (6)
C8—H8A	0.97	C23—O4	1.247 (6)
C8—H8B	0.97	N19—N20	1.343 (5)
C9—N9	1.462 (7)	N20—H20	0.86
C9—C10	1.501 (8)	C24—O5	1.235 (6)
C9—H9A	0.97	C24—O6	1.262 (5)
C9—H9B	0.97	C24—C25	1.483 (7)
C10—N10	1.475 (6)	C25—N21	1.337 (5)
C10—H10A	0.97	C25—C26	1.413 (6)
C10—H10B	0.97	C26—C27	1.367 (6)
C11—N11	1.471 (6)	C26—H26	0.93
C11—C12	1.504 (7)	C27—N22	1.353 (5)
C11—H11A	0.97	C27—C28	1.490 (7)
C11—H11B	0.97	C28—O7	1.236 (6)
C12—N12	1.466 (6)	C28—O8	1.246 (6)
C12—H12A	0.97	N21—N22	1.342 (5)
C12—H12B	0.97	N22—H22	0.86
N7—Ni2	2.106 (4)	C29—O10	1.202 (8)
N7—H7C	0.90	C29—O9	1.266 (8)
N7—H7D	0.90	C29—C30	1.465 (9)
N8—Ni2	2.130 (4)	C30—N23	1.342 (8)
N8—H8C	0.90	C30—C31	1.368 (8)
N8—H8D	0.90	C31—C32	1.397 (7)
N9—Ni2	2.124 (4)	C31—H31	0.93
N9—H9C	0.90	C32—N24	1.341 (7)
N9—H9D	0.90	C32—C33	1.497 (7)
N10—Ni2	2.125 (5)	C33—O11	1.246 (7)
N10—H10C	0.90	C33—O12	1.266 (7)
N10—H10D	0.90	N23—N24	1.358 (6)
N11—Ni2	2.126 (4)	N23—H23	0.8600
N11—H11C	0.90	O1W—H1W1	0.82 (5)
N11—H11D	0.90	O1W—H2W1	0.83 (8)
N12—Ni2	2.125 (4)	O2W—H1W2	0.82 (7)
N12—H12C	0.90	O2W—H2W2	0.83 (6)
N12—H12D	0.90	O3W—H1W3	0.84 (6)
C13—N13	1.472 (6)	O3W—H2W3	0.84 (6)
C13—C14	1.505 (7)	O4W—H1W4	0.83 (8)
C13—H13A	0.97	O4W—H2W4	0.84 (7)
C13—H13B	0.97	O5W—H1W5	0.830 (19)
C14—N14	1.477 (7)	O5W—H2W5	0.84 (4)
			
N1—C1—C2	109.1 (4)	N7—Ni2—N10	92.46 (18)
N1—C1—H1A	109.9	N9—Ni2—N10	81.63 (16)
C2—C1—H1A	109.9	N12—Ni2—N10	96.23 (17)
N1—C1—H1B	109.9	N7—Ni2—N11	167.90 (16)
C2—C1—H1B	109.9	N9—Ni2—N11	94.38 (15)
H1A—C1—H1B	108.3	N12—Ni2—N11	81.74 (14)
N2—C2—C1	108.1 (4)	N10—Ni2—N11	95.75 (18)
N2—C2—H2A	110.1	N7—Ni2—N8	82.19 (17)
C1—C2—H2A	110.1	N9—Ni2—N8	89.41 (16)
N2—C2—H2B	110.1	N12—Ni2—N8	93.14 (16)
C1—C2—H2B	110.1	N10—Ni2—N8	169.09 (15)
H2A—C2—H2B	108.4	N11—Ni2—N8	91.10 (17)
N3—C3—C4	110.2 (5)	N13—C13—C14	108.6 (4)
N3—C3—H3A	109.6	N13—C13—H13A	110.0
C4—C3—H3A	109.6	C14—C13—H13A	110.0
N3—C3—H3B	109.6	N13—C13—H13B	110.0
C4—C3—H3B	109.6	C14—C13—H13B	110.0
H3A—C3—H3B	108.1	H13A—C13—H13B	108.4
N4—C4—C3	107.6 (5)	N14—C14—C13	108.0 (4)
N4—C4—H4A	110.2	N14—C14—H14A	110.1
C3—C4—H4A	110.2	C13—C14—H14A	110.1
N4—C4—H4B	110.2	N14—C14—H14B	110.1
C3—C4—H4B	110.2	C13—C14—H14B	110.1
H4A—C4—H4B	108.5	H14A—C14—H14B	108.4
N5—C5—C6	108.7 (4)	N15—C15—C16	107.8 (5)
N5—C5—H5A	109.9	N15—C15—H15A	110.1
C6—C5—H5A	109.9	C16—C15—H15A	110.1
N5—C5—H5B	109.9	N15—C15—H15B	110.1
C6—C5—H5B	109.9	C16—C15—H15B	110.1
H5A—C5—H5B	108.3	H15A—C15—H15B	108.5
N6—C6—C5	108.6 (4)	N16—C16—C15	108.1 (5)
N6—C6—H6A	110.0	N16—C16—H16A	110.1
C5—C6—H6A	110.0	C15—C16—H16A	110.1
N6—C6—H6B	110.0	N16—C16—H16B	110.1
C5—C6—H6B	110.0	C15—C16—H16B	110.1
H6A—C6—H6B	108.4	H16A—C16—H16B	108.4
C1—N1—Ni1	107.1 (3)	N17—C17—C18	108.0 (4)
C1—N1—H1C	110.3	N17—C17—H17A	110.1
Ni1—N1—H1C	110.3	C18—C17—H17A	110.1
C1—N1—H1D	110.3	N17—C17—H17B	110.1
Ni1—N1—H1D	110.3	C18—C17—H17B	110.1
H1C—N1—H1D	108.6	H17A—C17—H17B	108.4
C2—N2—Ni1	108.4 (3)	N18—C18—C17	109.8 (4)
C2—N2—H2C	110.0	N18—C18—H18A	109.7
Ni1—N2—H2C	110.0	C17—C18—H18A	109.7
C2—N2—H2D	110.0	N18—C18—H18B	109.7
Ni1—N2—H2D	110.0	C17—C18—H18B	109.7
H2C—N2—H2D	108.4	H18A—C18—H18B	108.2
C3—N3—Ni1	108.4 (3)	C13—N13—Ni3	107.7 (3)
C3—N3—H3C	110.0	C13—N13—H13C	110.2
Ni1—N3—H3C	110.0	Ni3—N13—H13C	110.2
C3—N3—H3D	110.0	C13—N13—H13D	110.2
Ni1—N3—H3D	110.0	Ni3—N13—H13D	110.2
H3C—N3—H3D	108.4	H13C—N13—H13D	108.5
C4—N4—Ni1	108.7 (3)	C14—N14—Ni3	107.5 (3)
C4—N4—H4C	110.0	C14—N14—H14C	110.2
Ni1—N4—H4C	110.0	Ni3—N14—H14C	110.2
C4—N4—H4D	110.0	C14—N14—H14D	110.2
Ni1—N4—H4D	110.0	Ni3—N14—H14D	110.2
H4C—N4—H4D	108.3	H14C—N14—H14D	108.5
C5—N5—Ni1	109.3 (3)	C15—N15—Ni3	108.7 (4)
C5—N5—H5C	109.8	C15—N15—H15C	109.9
Ni1—N5—H5C	109.8	Ni3—N15—H15C	109.9
C5—N5—H5D	109.8	C15—N15—H15D	109.9
Ni1—N5—H5D	109.8	Ni3—N15—H15D	109.9
H5C—N5—H5D	108.3	H15C—N15—H15D	108.3
C6—N6—Ni1	107.8 (3)	C16—N16—Ni3	106.9 (3)
C6—N6—H6C	110.1	C16—N16—H16C	110.3
Ni1—N6—H6C	110.1	Ni3—N16—H16C	110.3
C6—N6—H6D	110.1	C16—N16—H16D	110.3
Ni1—N6—H6D	110.1	Ni3—N16—H16D	110.3
H6C—N6—H6D	108.5	H16C—N16—H16D	108.6
N4—Ni1—N2	95.88 (17)	C17—N17—Ni3	106.3 (3)
N4—Ni1—N5	90.55 (16)	C17—N17—H17C	110.5
N2—Ni1—N5	172.19 (18)	Ni3—N17—H17C	110.5
N4—Ni1—N6	171.11 (14)	C17—N17—H17D	110.5
N2—Ni1—N6	92.32 (17)	Ni3—N17—H17D	110.5
N5—Ni1—N6	81.55 (16)	H17C—N17—H17D	108.7
N4—Ni1—N3	81.46 (18)	C18—N18—Ni3	107.1 (3)
N2—Ni1—N3	91.30 (16)	C18—N18—H18C	110.3
N5—Ni1—N3	94.04 (15)	Ni3—N18—H18C	110.3
N6—Ni1—N3	94.97 (19)	C18—N18—H18D	110.3
N4—Ni1—N1	89.94 (17)	Ni3—N18—H18D	110.3
N2—Ni1—N1	82.15 (15)	H18C—N18—H18D	108.6
N5—Ni1—N1	93.46 (15)	N15—Ni3—N16	81.65 (19)
N6—Ni1—N1	94.56 (18)	N15—Ni3—N14	94.13 (17)
N3—Ni1—N1	168.64 (17)	N16—Ni3—N14	93.26 (18)
N7—C7—C8	110.1 (4)	N15—Ni3—N13	172.13 (18)
N7—C7—H7A	109.6	N16—Ni3—N13	91.78 (18)
C8—C7—H7A	109.6	N14—Ni3—N13	81.87 (16)
N7—C7—H7B	109.6	N15—Ni3—N17	94.52 (17)
C8—C7—H7B	109.6	N16—Ni3—N17	93.09 (18)
H7A—C7—H7B	108.2	N14—Ni3—N17	169.93 (19)
N8—C8—C7	108.2 (4)	N13—Ni3—N17	90.13 (16)
N8—C8—H8A	110.1	N15—Ni3—N18	90.18 (19)
C7—C8—H8A	110.1	N16—Ni3—N18	170.30 (17)
N8—C8—H8B	110.1	N14—Ni3—N18	92.52 (18)
C7—C8—H8B	110.1	N13—Ni3—N18	96.74 (19)
H8A—C8—H8B	108.4	N17—Ni3—N18	82.31 (18)
N9—C9—C10	108.9 (4)	O1—C19—O2	125.7 (5)
N9—C9—H9A	109.9	O1—C19—C20	116.6 (4)
C10—C9—H9A	109.9	O2—C19—C20	117.7 (4)
N9—C9—H9B	109.9	N19—C20—C21	110.8 (4)
C10—C9—H9B	109.9	N19—C20—C19	120.6 (4)
H9A—C9—H9B	108.3	C21—C20—C19	128.5 (4)
N10—C10—C9	109.0 (4)	C22—C21—C20	106.2 (4)
N10—C10—H10A	109.9	C22—C21—H21	126.9
C9—C10—H10A	109.9	C20—C21—H21	126.9
N10—C10—H10B	109.9	N20—C22—C21	105.2 (4)
C9—C10—H10B	109.9	N20—C22—C23	120.1 (4)
H10A—C10—H10B	108.3	C21—C22—C23	134.7 (4)
N11—C11—C12	109.2 (4)	O3—C23—O4	126.5 (5)
N11—C11—H11A	109.8	O3—C23—C22	116.9 (4)
C12—C11—H11A	109.8	O4—C23—C22	116.6 (5)
N11—C11—H11B	109.8	C20—N19—N20	104.6 (4)
C12—C11—H11B	109.8	N19—N20—C22	113.1 (4)
H11A—C11—H11B	108.3	N19—N20—H20	123.4
N12—C12—C11	107.9 (4)	C22—N20—H20	123.4
N12—C12—H12A	110.1	O5—C24—O6	124.2 (5)
C11—C12—H12A	110.1	O5—C24—C25	119.2 (4)
N12—C12—H12B	110.1	O6—C24—C25	116.5 (4)
C11—C12—H12B	110.1	N21—C25—C26	109.9 (4)
H12A—C12—H12B	108.4	N21—C25—C24	121.2 (4)
C7—N7—Ni2	109.3 (3)	C26—C25—C24	128.9 (4)
C7—N7—H7C	109.8	C27—C26—C25	106.0 (4)
Ni2—N7—H7C	109.8	C27—C26—H26	127.0
C7—N7—H7D	109.8	C25—C26—H26	127.0
Ni2—N7—H7D	109.8	N22—C27—C26	105.9 (4)
H7C—N7—H7D	108.3	N22—C27—C28	120.3 (4)
C8—N8—Ni2	106.3 (3)	C26—C27—C28	133.8 (4)
C8—N8—H8C	110.5	O7—C28—O8	125.3 (5)
Ni2—N8—H8C	110.5	O7—C28—C27	118.3 (5)
C8—N8—H8D	110.5	O8—C28—C27	116.4 (5)
Ni2—N8—H8D	110.5	C25—N21—N22	105.3 (4)
H8C—N8—H8D	108.7	N21—N22—C27	112.8 (4)
C9—N9—Ni2	107.4 (3)	N21—N22—H22	123.6
C9—N9—H9C	110.2	C27—N22—H22	123.6
Ni2—N9—H9C	110.2	O10—C29—O9	123.3 (6)
C9—N9—H9D	110.2	O10—C29—C30	121.2 (7)
Ni2—N9—H9D	110.2	O9—C29—C30	115.4 (6)
H9C—N9—H9D	108.5	N23—C30—C31	105.5 (5)
C10—N10—Ni2	108.5 (3)	N23—C30—C29	120.0 (6)
C10—N10—H10C	110.0	C31—C30—C29	134.5 (7)
Ni2—N10—H10C	110.0	C30—C31—C32	106.1 (5)
C10—N10—H10D	110.0	C30—C31—H31	126.9
Ni2—N10—H10D	110.0	C32—C31—H31	126.9
H10C—N10—H10D	108.4	N24—C32—C31	111.2 (4)
C11—N11—Ni2	106.8 (3)	N24—C32—C33	120.1 (5)
C11—N11—H11C	110.4	C31—C32—C33	128.7 (5)
Ni2—N11—H11C	110.4	O11—C33—O12	126.3 (5)
C11—N11—H11D	110.4	O11—C33—C32	118.0 (6)
Ni2—N11—H11D	110.4	O12—C33—C32	115.7 (5)
H11C—N11—H11D	108.6	C30—N23—N24	113.9 (5)
C12—N12—Ni2	108.4 (3)	C30—N23—H23	123.0
C12—N12—H12C	110.0	N24—N23—H23	123.0
Ni2—N12—H12C	110.0	C32—N24—N23	103.2 (4)
C12—N12—H12D	110.0	H1W1—O1W—H2W1	110 (8)
Ni2—N12—H12D	110.0	H1W2—O2W—H2W2	110 (7)
H12C—N12—H12D	108.4	H1W3—O3W—H2W3	108 (3)
N7—Ni2—N9	95.60 (15)	H1W4—O4W—H2W4	108 (7)
N7—Ni2—N12	88.56 (15)	H1W5—O5W—H2W5	107 (3)
N9—Ni2—N12	175.39 (16)		
			
N1—C1—C2—N2	56.5 (6)	C18—C17—N17—Ni3	−45.2 (4)
N3—C3—C4—N4	54.5 (5)	C17—C18—N18—Ni3	−39.3 (5)
N5—C5—C6—N6	54.8 (6)	C15—N15—Ni3—N16	13.0 (4)
C2—C1—N1—Ni1	−41.4 (5)	C15—N15—Ni3—N14	−79.7 (4)
C1—C2—N2—Ni1	−41.9 (5)	C15—N15—Ni3—N17	105.5 (4)
C4—C3—N3—Ni1	−38.2 (5)	C15—N15—Ni3—N18	−172.2 (4)
C3—C4—N4—Ni1	−42.6 (5)	C16—N16—Ni3—N15	17.6 (4)
C6—C5—N5—Ni1	−37.3 (5)	C16—N16—Ni3—N14	111.3 (4)
C5—C6—N6—Ni1	−44.0 (5)	C16—N16—Ni3—N13	−166.8 (4)
C4—N4—Ni1—N2	108.0 (3)	C16—N16—Ni3—N17	−76.6 (4)
C4—N4—Ni1—N5	−76.4 (3)	C14—N14—Ni3—N15	−170.9 (4)
C4—N4—Ni1—N3	17.6 (3)	C14—N14—Ni3—N16	107.2 (4)
C4—N4—Ni1—N1	−169.9 (3)	C14—N14—Ni3—N13	15.9 (4)
C2—N2—Ni1—N4	104.3 (4)	C14—N14—Ni3—N17	−21.8 (12)
C2—N2—Ni1—N6	−79.1 (4)	C14—N14—Ni3—N18	−80.6 (4)
C2—N2—Ni1—N3	−174.1 (4)	C13—N13—Ni3—N16	−78.8 (4)
C2—N2—Ni1—N1	15.2 (4)	C13—N13—Ni3—N14	14.2 (4)
C5—N5—Ni1—N4	−173.5 (3)	C13—N13—Ni3—N17	−171.9 (4)
C5—N5—Ni1—N6	10.6 (3)	C13—N13—Ni3—N18	105.8 (4)
C5—N5—Ni1—N3	105.0 (4)	C17—N17—Ni3—N15	108.4 (3)
C5—N5—Ni1—N1	−83.5 (4)	C17—N17—Ni3—N16	−169.8 (3)
C6—N6—Ni1—N2	−166.5 (3)	C17—N17—Ni3—N14	−40.7 (12)
C6—N6—Ni1—N5	18.4 (3)	C17—N17—Ni3—N13	−78.0 (3)
C6—N6—Ni1—N3	−75.0 (3)	C17—N17—Ni3—N18	18.8 (3)
C6—N6—Ni1—N1	111.2 (3)	C18—N18—Ni3—N15	−83.6 (3)
C3—N3—Ni1—N4	11.2 (3)	C18—N18—Ni3—N14	−177.7 (3)
C3—N3—Ni1—N2	−84.6 (3)	C18—N18—Ni3—N13	100.2 (3)
C3—N3—Ni1—N5	101.1 (3)	C18—N18—Ni3—N17	11.0 (3)
C3—N3—Ni1—N6	−177.0 (3)	O1—C19—C20—N19	−177.5 (4)
C3—N3—Ni1—N1	−30.1 (10)	O2—C19—C20—N19	1.3 (7)
C1—N1—Ni1—N4	−81.6 (4)	O1—C19—C20—C21	5.4 (8)
C1—N1—Ni1—N2	14.3 (4)	O2—C19—C20—C21	−175.8 (5)
C1—N1—Ni1—N5	−172.2 (4)	N19—C20—C21—C22	−0.1 (6)
C1—N1—Ni1—N6	106.1 (4)	C19—C20—C21—C22	177.2 (5)
C1—N1—Ni1—N3	−40.9 (10)	C20—C21—C22—N20	−0.3 (6)
N7—C7—C8—N8	−54.6 (5)	C20—C21—C22—C23	180.0 (6)
N9—C9—C10—N10	−55.8 (6)	N20—C22—C23—O3	−7.8 (8)
N11—C11—C12—N12	−57.5 (6)	C21—C22—C23—O3	171.9 (6)
C8—C7—N7—Ni2	34.9 (4)	N20—C22—C23—O4	172.7 (5)
C7—C8—N8—Ni2	45.1 (4)	C21—C22—C23—O4	−7.6 (9)
C10—C9—N9—Ni2	44.4 (5)	C21—C20—N19—N20	0.4 (5)
C9—C10—N10—Ni2	37.7 (5)	C19—C20—N19—N20	−177.1 (4)
C12—C11—N11—Ni2	43.8 (5)	C20—N19—N20—C22	−0.6 (6)
C11—C12—N12—Ni2	40.6 (5)	C21—C22—N20—N19	0.6 (6)
C7—N7—Ni2—N9	−96.4 (3)	C23—C22—N20—N19	−179.7 (4)
C7—N7—Ni2—N12	85.6 (3)	O5—C24—C25—N21	5.4 (8)
C7—N7—Ni2—N10	−178.2 (3)	O6—C24—C25—N21	−173.2 (5)
C7—N7—Ni2—N11	49.1 (9)	O5—C24—C25—C26	−173.1 (5)
C7—N7—Ni2—N8	−7.7 (3)	O6—C24—C25—C26	8.3 (8)
C9—N9—Ni2—N7	−110.1 (3)	N21—C25—C26—C27	0.7 (6)
C9—N9—Ni2—N10	−18.4 (3)	C24—C25—C26—C27	179.4 (5)
C9—N9—Ni2—N11	76.8 (3)	C25—C26—C27—N22	−0.6 (6)
C9—N9—Ni2—N8	167.9 (3)	C25—C26—C27—C28	−177.7 (6)
C12—N12—Ni2—N7	173.8 (4)	N22—C27—C28—O7	−171.1 (5)
C12—N12—Ni2—N10	81.5 (4)	C26—C27—C28—O7	5.6 (9)
C12—N12—Ni2—N11	−13.4 (4)	N22—C27—C28—O8	7.7 (8)
C12—N12—Ni2—N8	−104.1 (4)	C26—C27—C28—O8	−175.6 (6)
C10—N10—Ni2—N7	84.6 (3)	C26—C25—N21—N22	−0.5 (6)
C10—N10—Ni2—N9	−10.7 (3)	C24—C25—N21—N22	−179.3 (5)
C10—N10—Ni2—N12	173.4 (3)	C25—N21—N22—C27	0.1 (6)
C10—N10—Ni2—N11	−104.3 (3)	C26—C27—N22—N21	0.3 (6)
C10—N10—Ni2—N8	24.3 (11)	C28—C27—N22—N21	177.9 (5)
C11—N11—Ni2—N7	20.5 (10)	O10—C29—C30—N23	12.0 (11)
C11—N11—Ni2—N9	166.0 (3)	O9—C29—C30—N23	−165.3 (6)
C11—N11—Ni2—N12	−16.5 (3)	O10—C29—C30—C31	−166.6 (7)
C11—N11—Ni2—N10	−112.0 (3)	O9—C29—C30—C31	16.1 (12)
C11—N11—Ni2—N8	76.5 (3)	N23—C30—C31—C32	−0.2 (7)
C8—N8—Ni2—N7	−20.8 (3)	C29—C30—C31—C32	178.6 (8)
C8—N8—Ni2—N9	74.9 (3)	C30—C31—C32—N24	0.5 (6)
C8—N8—Ni2—N12	−108.9 (3)	C30—C31—C32—C33	179.9 (5)
C8—N8—Ni2—N10	40.3 (10)	N24—C32—C33—O11	−18.3 (7)
C8—N8—Ni2—N11	169.3 (3)	C31—C32—C33—O11	162.3 (5)
N13—C13—C14—N14	57.6 (6)	N24—C32—C33—O12	162.4 (4)
N15—C15—C16—N16	58.0 (6)	C31—C32—C33—O12	−17.0 (7)
N17—C17—C18—N18	58.2 (6)	C31—C30—N23—N24	−0.2 (7)
C14—C13—N13—Ni3	−41.8 (5)	C29—C30—N23—N24	−179.2 (6)
C13—C14—N14—Ni3	−43.0 (5)	C31—C32—N24—N23	−0.6 (5)
C16—C15—N15—Ni3	−40.8 (6)	C33—C32—N24—N23	179.9 (4)
C15—C16—N16—Ni3	−44.7 (6)	C30—N23—N24—C32	0.5 (6)

### Hydrogen-bond geometry (Å, °)

**Table:**

*D*—H···*A*	*D*—H	H···*A*	*D*···*A*	*D*—H···*A*
O1W—H1W1···O4	0.82 (5)	1.93 (3)	2.725 (6)	162 (8)
O1W—H2W1···O9	0.83 (8)	1.94 (8)	2.739 (8)	163 (8)
O2W—H1W2···O6	0.82 (7)	1.90 (3)	2.674 (5)	159 (6)
O2W—H2W2···O1	0.83 (6)	2.04 (3)	2.856 (6)	167 (8)
O3W—H1W3···O7	0.84 (6)	1.95 (6)	2.787 (6)	175 (2)
O3W—H2W3···O11^i^	0.84 (6)	2.36 (4)	3.074 (6)	144 (6)
O3W—H2W3···N24^i^	0.84 (6)	2.55 (5)	3.236 (6)	141 (7)
O4W—H1W4···O1W^ii^	0.83 (8)	2.37 (5)	3.094 (8)	146 (8)
O4W—H1W4···O9^ii^	0.83 (8)	2.57 (5)	3.293 (6)	146 (8)
O4W—H2W4···O5W	0.84 (7)	2.03 (7)	2.839 (6)	163 (7)
O5W—H1W5···O12^iii^	0.83 (2)	2.01 (3)	2.819 (5)	165 (6)
O5W—H2W5···O12^ii^	0.84 (4)	2.06 (2)	2.888 (5)	176 (7)
N1—H1C···O1^ii^	0.90	2.34	3.215 (6)	164
N1—H1D···O3W^ii^	0.90	2.27	3.159 (7)	170
N2—H2C···O9^iv^	0.90	2.25	3.118 (6)	162
N2—H2D···O1^iv^	0.90	2.55	3.358 (6)	150
N3—H3D···N21^v^	0.90	2.24	3.066 (5)	153
N4—H4C···O2^ii^	0.90	2.24	3.091 (5)	158
N4—H4D···O1^iv^	0.90	2.15	3.039 (5)	168
N5—H5C···O2^ii^	0.90	2.28	3.091 (6)	150
N5—H5D···O5^v^	0.90	2.02	2.913 (5)	174
N6—H6C···O10^iv^	0.90	2.38	3.154 (6)	144
N6—H6C···O9^iv^	0.90	2.54	3.357 (6)	152
N7—H7C···N19^vi^	0.90	2.07	2.950 (5)	165
N7—H7D···O3W	0.90	2.45	3.314 (6)	162
N8—H8C···O2W	0.90	2.22	3.029 (6)	149
N8—H8D···O5^vii^	0.90	2.57	3.388 (6)	152
N8—H8D···O6^vii^	0.90	2.59	3.345 (5)	142
N9—H9C···O2^vi^	0.90	2.19	3.071 (5)	166
N9—H9D···O5^vii^	0.90	2.18	3.049 (6)	162
N10—H10D···O11^i^	0.90	2.21	3.066 (6)	159
N11—H11C···O1W^vii^	0.90	2.33	3.173 (7)	156
N11—H11D···O6^vii^	0.90	2.09	2.959 (6)	162
N12—H12C···O2W	0.90	2.22	3.100 (7)	166
N12—H12D···O3W	0.90	2.46	3.213 (6)	142
N13—H13C···O8^viii^	0.90	2.08	2.929 (6)	157
N13—H13D···O7	0.90	2.57	3.307 (7)	139
N14—H14C···O4^vii^	0.90	2.08	2.976 (6)	173
N14—H14D···O12^vii^	0.90	2.41	3.284 (7)	163
N15—H15C···O3^vii^	0.90	2.13	2.940 (6)	149
N16—H16C···O8^viii^	0.90	2.20	3.017 (6)	151
N16—H16D···O12^vii^	0.90	2.27	3.147 (5)	164
N17—H17C···O7^viii^	0.90	2.42	3.290 (6)	162
N17—H17C···O8^viii^	0.90	2.59	3.351 (6)	142
N17—H17D···O10^ix^	0.90	2.31	3.151 (6)	155
N18—H18C···O3^vii^	0.90	2.25	3.100 (6)	157
N20—H20···O11^x^	0.86	2.35	3.058 (6)	140
N22—H22···O4W	0.86	2.00	2.819 (5)	160
N23—H23···O10^xi^	0.86	2.03	2.856 (6)	160

Symmetry codes: (i) *x*, *y*, *z*−1; (ii) −*x*+1, −*y*+1, −*z*+1; (iii) *x*+1, *y*, *z*−1; (iv) *x*, *y*+1, *z*; (v) −*x*+1, −*y*+2, −*z*+1; (vi) −*x*, −*y*, −*z*+1; (vii) −*x*, −*y*+1, −*z*+1; (viii) −*x*+1, −*y*+1, −*z*; (ix) *x*, *y*+1, *z*−1; (x) −*x*, −*y*, −*z*+2; (xi) −*x*+1, −*y*, −*z*+2.
